# The impact of mobile health interventions on patients with schizophrenia: a meta-analysis

**DOI:** 10.3389/fpsyt.2025.1715324

**Published:** 2026-01-05

**Authors:** Yongli Ye, Fangrui Zhang, Yangyang Zhou

**Affiliations:** 1 Seventh Ward, Lishui Second People’s Hospital, Lishui, Zhejiang, China; 2 Department of Psychiatric, Ningbo Kangning Hospital, Ningbo, Zhejiang, China; 3 Department of Material Dependence, Lishui Second People’s Hospital, Lishui, Zhejiang, China

**Keywords:** depression, incidence of adverse events, meta-analysis, mobile health, positive symptoms, schizophrenia

## Abstract

**Objective:**

To systematically evaluate the impact of mobile health (mHealth) interventions on patients with schizophrenia.

**Methods:**

Randomized controlled trials (RCTs) concerning the effects of mHealth interventions on patients with schizophrenia were retrieved from databases including CNKI, WanFang, VIP, CBM, PubMed, Cochrane Library, Embase, and Web of Science from their inception until September 2025. Data analysis was performed using RevMan 5.4 software.

**Results:**

A total of 10 RCTs involving 1135 patients with schizophrenia were included. The analysis results showed that the intervention group was significantly better than the control group in improving positive symptoms of schizophrenia (*SMD* = -0.18, *95% CI* = -0.34 to -0.02, *P* = 0.03). The control group had a lower incidence of adverse events compared to the intervention group (*OR* = 2.30, *95% CI* = 1.31 to 4.04, *P* = 0.004). There was no significant difference between the two groups in depressive symptoms (*SMD* = -0.02, *95% CI* = -0.23 to 0.19, *P* = 0.83).

**Conclusion:**

mHealth interventions demonstrate a slight but definite positive effect on improving positive symptoms in schizophrenia. However, they show no significant effect on improving depressive symptoms. While a higher incidence of adverse events was reported in the intervention group, this finding should be interpreted with caution as it may be influenced by enhanced detection through digital monitoring (surveillance bias).

## Introduction

Schizophrenia is a chronic and severe mental disorder with high disability rates. Its core positive symptoms (such as hallucinations and delusions), along with accompanying negative symptoms and cognitive deficits, impose a substantial disease burden on patients, families, and society (1). Although antipsychotic medications form the cornerstone of treatment, a considerable proportion of patients experience issues such as suboptimal efficacy and adverse drug effects, leading to poor treatment adherence and an increased risk of relapse. Furthermore, depressive symptoms are highly prevalent among individuals with schizophrenia, with a comorbidity rate as high as 50%, representing a significant factor associated with reduced quality of life, functional disability, and elevated suicide risk (2). Consequently, developing effective and accessible adjuvant interventions to synergistically manage both psychotic symptoms and depressive mood constitutes a key challenge in contemporary mental health services.

In recent years, with the rapid advancement of digital health, mobile health (mHealth) has emerged as a novel intervention model offering new opportunities to improve the long-term management of schizophrenia. mHealth interventions broadly refer to health support services delivered via mobile devices such as smartphones and tablets, encompassing various formats including remote symptom monitoring, self-help courses based on cognitive behavioral therapy, medication reminders, and provision of social support (3). Compared to traditional face-to-face interventions, mHealth offers unique advantages such as transcending geographical and temporal constraints, strong anonymity, and the capacity for real-time intervention. These features are particularly appealing for patients who face barriers to consistent in-person services due to stigma, geographical distance, or physical limitations (4). Although several preliminary studies have explored the efficacy of mHealth interventions for patients with schizophrenia, the existing evidence presents inconsistent conclusions. Some studies indicate that mHealth can significantly improve positive symptoms and depression, while others have found it not significantly superior to treatment as usual (5). Therefore, this meta-analysis aims to synthesize the impact of mHealth interventions on patients with schizophrenia. The findings will provide critical evidence for clinicians to develop evidence-based intervention strategies, for researchers to plan future research directions, and for public health policymakers to optimize the allocation of mental health service resources.

## Methods

### Inclusion and exclusion criteria

The inclusion criteria for the literature were as follows. (1) Type of study: Randomized Controlled Trial (RCT). (2) Study subjects: Patients meeting the diagnostic criteria for schizophrenia as defined by the International Classification of Diseases, 10th Revision (ICD-10) (6), the Diagnostic and Statistical Manual of Mental Disorders, Fifth Edition (DSM-5) (7), or the Chinese Classification of Mental Disorders, Third Edition (CCMD-3) (8). (3) Interventions: The intervention group received mHealth interventions; the control group received usual care (traditional face-to-face interventions). (4) The primary outcomes were positive symptoms, depressive symptoms, and the incidence of adverse events. Included studies had to report at least one of these primary outcomes.

The exclusion criteria for the literature were as follows. (1) Duplicate publications; (2) Literature with incomplete relevant data; (3) Literature for which the full text was unavailable; (4) Non-English or non-Chinese literature.

### Search strategy

A comprehensive search was performed using a combination of subject headings (e.g., MeSH) and free-text terms. The search strategy was built around three concepts: (1) the population: “Schizophrenia” OR “psychotic disorders” OR “serious mental illness”; (2) the intervention: “Telemedicine” OR “Telepsychiatry” OR “Telehealth” OR “mHealth” OR “eHealth” OR “mobile health” OR “smartphone” OR “mobile applications” OR “mobile app” OR “text message” OR “SMS” OR “internet-based” OR “Web-based” OR “digital intervention” OR “ecological momentary assessment” OR “ecological momentary intervention”; and (3) the study design: the Cochrane Highly Sensitive Search Strategy for identifying randomized trials was applied where available. No restrictions on publication date were applied. The reference lists of included articles were reviewed for additional studies. Trial registries were not hand-searched. The search was restricted to studies published in English or Chinese.

### Literature screening and data extraction

Two researchers (Ye and Zhang) independently screened the literature based on the inclusion and exclusion criteria. After removing duplicate records, they reviewed the titles and abstracts, followed by a full-text assessment. In case of disagreement, a third researcher (Zhou) was consulted to make the final decision on inclusion. Data extraction was performed independently by two researchers using a standardized, pre-piloted data extraction form. To ensure accuracy, the process involved double data entry and verification; one researcher performed the initial extraction, and the second researcher cross-checked the entries against the original articles. Discrepancies were resolved by consensus or by adjudication from a third researcher. The extracted information included the title, author(s), country, year of publication, sample size, participant age, intervention details and content, intervention duration, and outcome measures. For studies with missing or ambiguous data, we attempted to contact the corresponding authors via email to request the required information. If standard deviations were missing and could not be obtained, they were imputed using a correlation coefficient derived from other complete studies within the review, following the Cochrane Handbook’s recommendations. The specific instruments used to assess outcomes in the included studies were extracted. As detailed in the following summary, positive symptoms were primarily measured using the Positive and Negative Syndrome Scale (PANSS) positive subscale or the Brief Psychiatric Rating Scale (BPRS). Depressive symptoms were assessed using scales such as the Calgary Depression Scale for Schizophrenia (CDSS), the Hamilton Depression Rating Scale (HAMD), or the Patient Health Questionnaire (PHQ-9). All studies that measured a given outcome used one of the instruments listed here; the specific instrument for each study is available from the authors upon reasonable request. Adverse events were reported as incidence counts. For the cluster-randomized trial included in this review (9), we utilized the analyzed data as reported by the trial authors. An adjustment for the design effect was not feasible due to the lack of reported intra-cluster correlation coefficients (ICCs) or the effective sample size. The potential impact of this on the overall results was considered in the interpretation of the findings and is acknowledged as a limitation.

### Quality assessment

The methodological quality of the included studies was assessed independently by two researchers using the Cochrane Risk of Bias Tool (RoB 2.0) (10). This tool evaluates the following five domains: (1) randomization process; (2) deviations from intended interventions; (3) missing outcome data; (4) outcome measurement; and (5) selective reporting of results. Each domain was judged as “low risk,” “some concerns,” or “high risk.” The overall risk of bias was determined based on the most severe rating across the domains. Two researchers independently conducted the assessments, and any disagreements were resolved through discussion or consultation with a third researcher.

### Evidence certainty assessment

For the three primary outcomes of this review (positive symptoms, incidence of adverse events, depressive symptoms), we will use the Grading of Recommendations Assessment, Development and Evaluation (GRADE) approach to assess the certainty of evidence (11). The GRADE assessment starts with high certainty for randomized trials and may be downgraded based on five domains: risk of bias, inconsistency, indirectness, imprecision, and publication bias. The certainty of evidence will be categorized as high, moderate, low, or very low.

### Statistical methods

Meta-analysis was performed using Review Manager (RevMan) software, version 5.4. The inverse-variance method was used for all meta-analyses. For quantitative data, the standardized mean difference (SMD) was used as the effect size, presented as point estimates with 95% confidence intervals (CI). Heterogeneity among the included studies was assessed using the I² statistic and the Tau² (Tau-squared) statistic. The I² statistic describes the percentage of total variation across studies that is due to heterogeneity rather than chance. The Tau² statistic estimates the between-study variance in a random-effects meta-analysis. A fixed-effect model (IV, Fixed) was applied when heterogeneity was considered non-significant (typically I² ≤ 50%). When significant heterogeneity was present (typically I² > 50%), a random-effects model (IV, Random) was employed to incorporate an estimate of the between-study variance into the analysis. If significant heterogeneity was detected, sensitivity analyses were conducted to explore the potential sources. For continuous outcomes, to ensure consistent interpretation across all scales, higher scores were uniformly aligned to represent greater symptom severity. Consequently, a negative Standardized Mean Difference (SMD) indicates a reduction in symptoms (i.e., improvement) favoring the intervention group. The specific model used for each outcome is reported in the results.

### Adverse events: definition and classification

In this review, an adverse event was defined as any untoward medical occurrence during the intervention period, regardless of its causal relationship to the intervention. We endeavored to extract the following information from the original studies for further distinction: (1) Type: categorized as psychiatric (e.g., exacerbated anxiety, relapse of psychotic symptoms), somatic (e.g., headache, insomnia), or technology-related (e.g., difficulty using the application, privacy concerns); (2) Severity: classified according to conventional clinical standards into serious adverse events (e.g., those leading to hospitalization, being life-threatening, or resulting in significant functional impairment) and non-serious adverse events (e.g., transient, mild discomfort). However, as most original studies did not provide such detailed categorical data, this analysis is primarily based on the overall incidence of adverse events reported by the individual studies.

## Results

### Basic characteristics and methodological quality assessment of included studies

**Figure 1:**
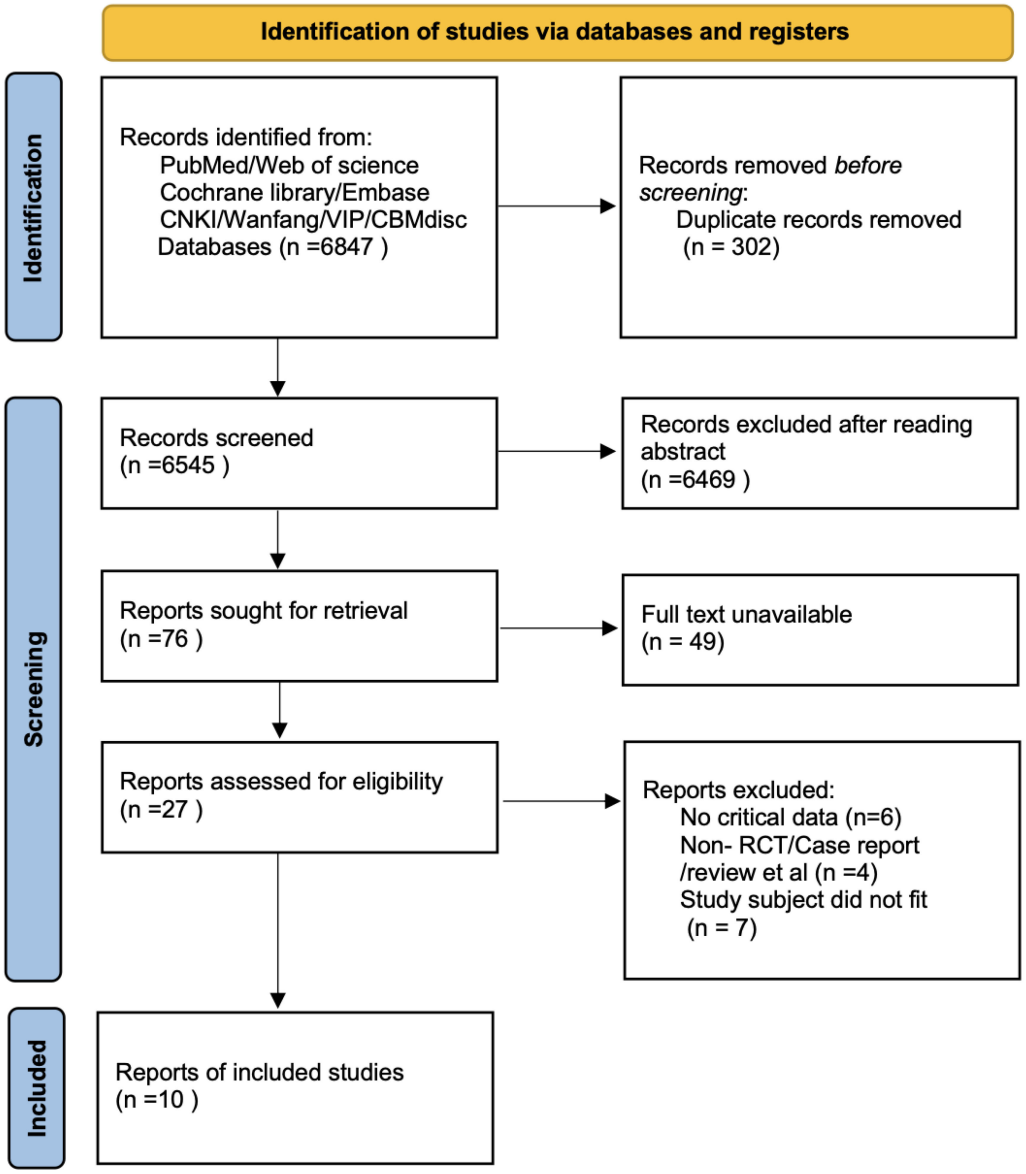
Literature screening flowchart.

**Figure 2:**
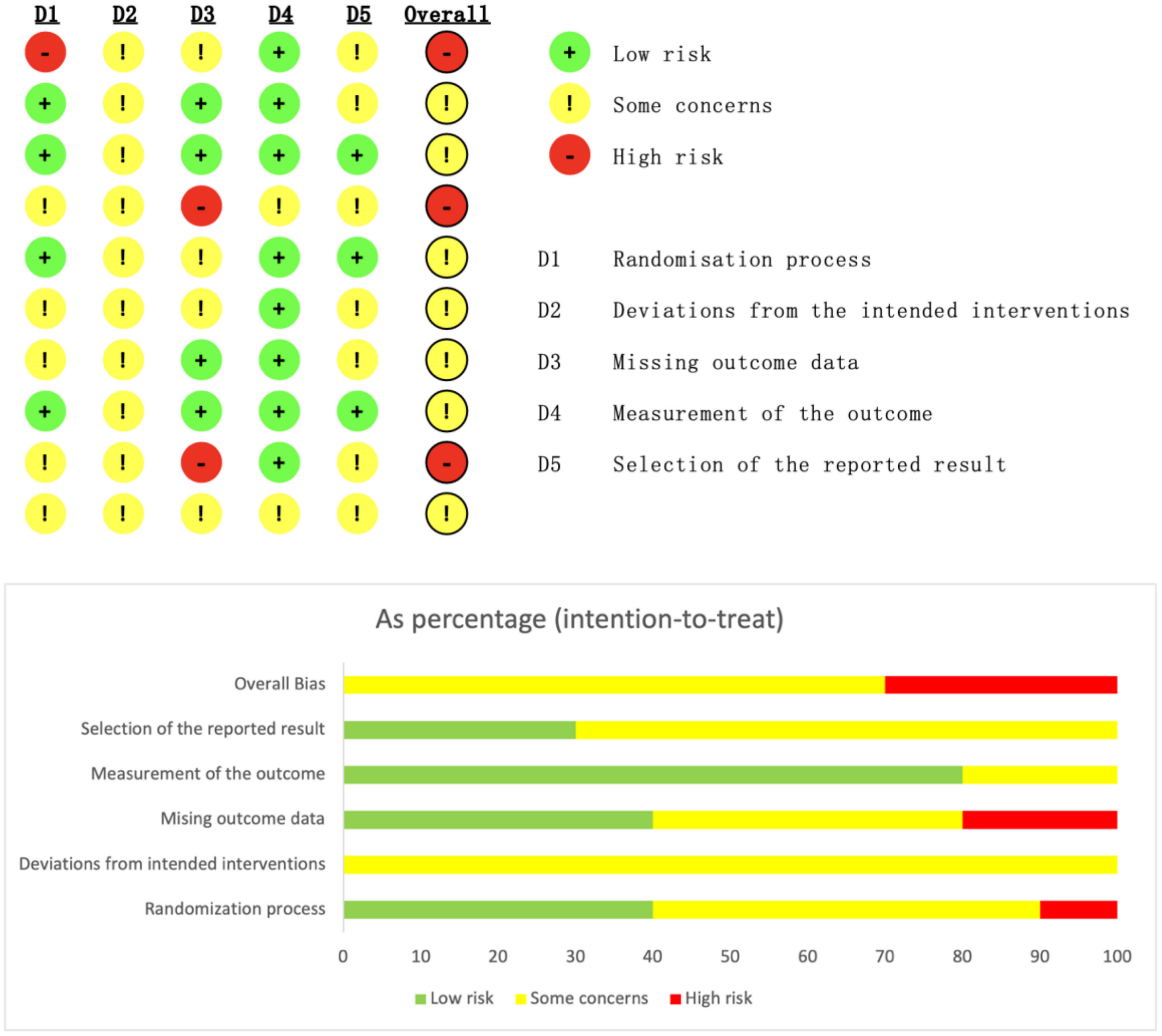
Risk of bias assessment graph. The bars represent the proportion of studies judged to have low, unclear, or high risk of bias for each domain: 1. Random sequence generation (selection bias); 2. Allocation concealment (selection bias); 3. Blinding of participants and personnel (performance bias); 4. Blinding of outcome assessment (detection bias); 5. Incomplete outcome data (attrition bias); 6. Selective reporting (reporting bias); 7. Other bias.

A total of 6,847 records were identified through database searching. Ultimately, 10 studies (9, 12–20) were included in the analysis. The literature screening process is illustrated in **Figure 1**. The basic characteristics of the included studies are presented in **Table 1**. The risk of bias assessment results for the included studies using the RoB 2.0 tool showed that 0 studies had an overall low risk of bias, 7 studies raised some concerns, and 3 studies had a high risk of bias. A summary of the risk of bias assessment results is shown in **Figure 2**. The risk of bias graph for the included studies is shown in **Figure 2**. The GRADE certainty of evidence assessments for the three primary outcomes is presented in **Table 2**. Overall, the certainty of evidence was low for improvement in positive symptoms and for increased risk of adverse events, and moderate for the lack of a significant effect on depressive symptoms.

**Table 1:** Basic characteristics of included studies.

Author and year	Country	Sample size		Age		Intervention method		Intervention duration	Outcome measures
		Control group	Intervention group	Control group	Intervention group	Intervention group	Control group		
Kimhy2025 (12)	USA	19	14	40.00 ± 10.00	33.00 ± 10.00	Remote aerobic exercise	Face-to-face aerobic exercise	12 weeks	③
Ben-Zeev2018 (13)	USA	70	74	NR	NR	Smartphone intervention	Usual care	12 weeks	②
Cai2022 (14)	China	128	128	NR	NR	LEAN intervention	Usual care	6 months	②③
Vitger2022 (15)	Denmark	98	96	24.30 ± 4.30	22.70 ± 3.70	Smartphone App	Usual care	6 months	①
Krzystanek2019 (16)	Poland	91	199	32.20 ± 6.94	32.00 ± 5.92	MONEO smartphone	Usual care	12 months	①②③
Flaherty2017 (17)	USA	25	20	51.20 ± 11.10	49.90 ± 12.70	Home telehealth	Usual care	3 months	②
Gumley2022 (9)	Australia	25	30	43.00 ± 12.00	42.00 ± 13.00	Smartphone APP	Usual care	12 months	②③
Frangou2005 (18)	UK	36	36	NR	NR	HOME platform	Usual care	8 weeks	②
Bucci2024 (19)	UK	76	68	NR	NR	Actissist app	Usual care	12 weeks	①②
Katsushima2025 (20)	Japan	12	12	32.30 ± 12.26	34.70 ± 8.98	Remote video cognitive behavioral therapy	Usual care	7 weeks	①②

① Positive symptoms of psychosis; ② Depression; ③ Incidence of adverse events.

**Table 2:** Summary of findings (GRADE): mobile health (mHealth) interventions for patients with schizophrenia.

Outcome	№ of participants (studies)	Certainty of the evidence (GRADE)	Anticipated absolute effects	
			Intervention group (95CI%)	Control group
Positive symptoms	652 (4 RCTs)	low^a,b^	SMD -0.18 (95% CI: -0.34 to -0.02)	–
Depressive symptoms	1026 (8 RCTs)	moderate^c^	SMD -0.02 (95% CI: -0.23 to 0.19)	–
Incidence of adverse events	634 (4 RCTs)	low^d,e^	187 per 1,000 OR 2.30 (1.31 to 4.04)	91.2 per 1,000

Explanations

a. Downgraded one level for risk of bias: Most included studies had ‘some concerns’ regarding blinding of participants and personnel (performance bias).

b. Downgraded one level for imprecision: The 95% confidence interval includes both a negligible effect and a small benefit, and the total sample size is less than the optimal information size.

c. Downgraded one level for inconsistency: Substantial statistical heterogeneity was observed (I² = 57%).

d. Downgraded one level for risk of bias: Lack of blinding may have influenced the reporting and detection of adverse events (detection bias).

e. Downgraded one level for imprecision: The confidence interval is wide and includes both appreciable benefit and harm, and the number of events is relatively small.

The risk in the intervention group (and its 95% confidence interval) is based on the assumed risk in the control group and the relative effect of the intervention (and its 95% CI).

The participant numbers (N) for each outcome represent the total sample size from the studies included in the respective meta-analysis.

### Meta-analysis results

#### Positive symptoms

**Figure 3:**
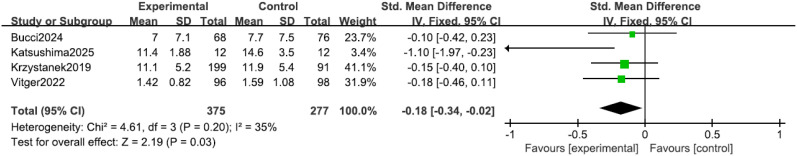
Forest plot of the meta-analysis for positive symptoms of psychosis.

All four studies (15, 16, 19, 20) reported positive symptoms in patients. Heterogeneity among the studies was low (I² = 35%, P = 0.20), therefore an inverse-variance fixed-effect (IV, Fixed) model was applied. The results indicated that the positive psychotic symptom scores in the intervention group were lower than those in the control group (SMD = -0.18, 95% CI = -0.34 to -0.02, P = 0.03), where the negative SMD favors the mHealth intervention, as shown in **Figure 3**. According to Cohen’s conventional criteria, this SMD of −0.18 represents a small effect size. To aid clinical interpretation, assuming a typical standard deviation between 5 and 8 points for the PANSS positive subscale, this corresponds to a reduction of approximately 1 to 1.5 points on that scale.

#### Incidence of adverse events

**Figure 4:**
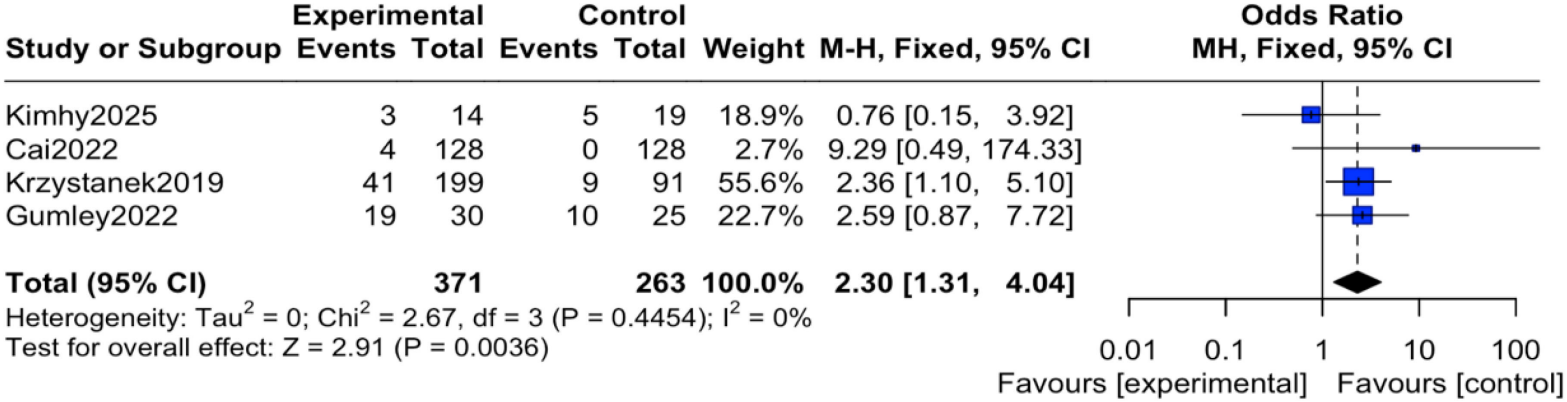
Forest plot of the meta-analysis for incidence of adverse events.

**Figure 5:**
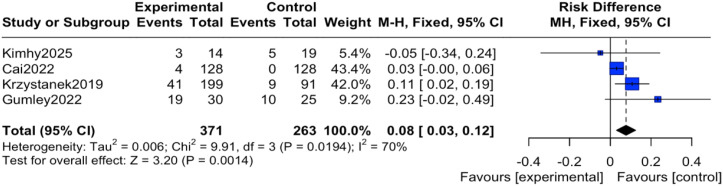
Forest plot of the meta-analysis for the incidence of adverse events (Risk Difference).

The reported events across these trials encompassed a range of outcomes. For instance, study (12) reported transient anxiety and sleep disturbances related to the exercise intervention; study (14) noted mild somatic discomfort; study (16) included events such as headache and agitation; and study (19) reported more serious events including hospitalization for psychiatric relapse. It is important to note that none of the studies documented adverse events directly attributable to the digital device or application itself (e.g., eye strain, digital-specific anxiety). Regarding the pooled analysis, there was no heterogeneity among the studies (I² = 0%, P = 0.45), therefore an inverse-variance fixed-effect (IV, Fixed) model was applied. The results indicated that the reported incidence of adverse events was significantly higher in the intervention group compared to the control group, with the difference being statistically significant (OR = 2.30, 95% CI = 1.31 to 4.04, P = 0.004), as shown in **Figure 4**. To express this risk in absolute terms, the risk difference (RD) was 0.08 (95% CI 0.03 to 0.12), indicating an absolute increase in risk of 8% in the mHealth group, as shown in **Figure 5**.

#### Depression

**Figure 6:**
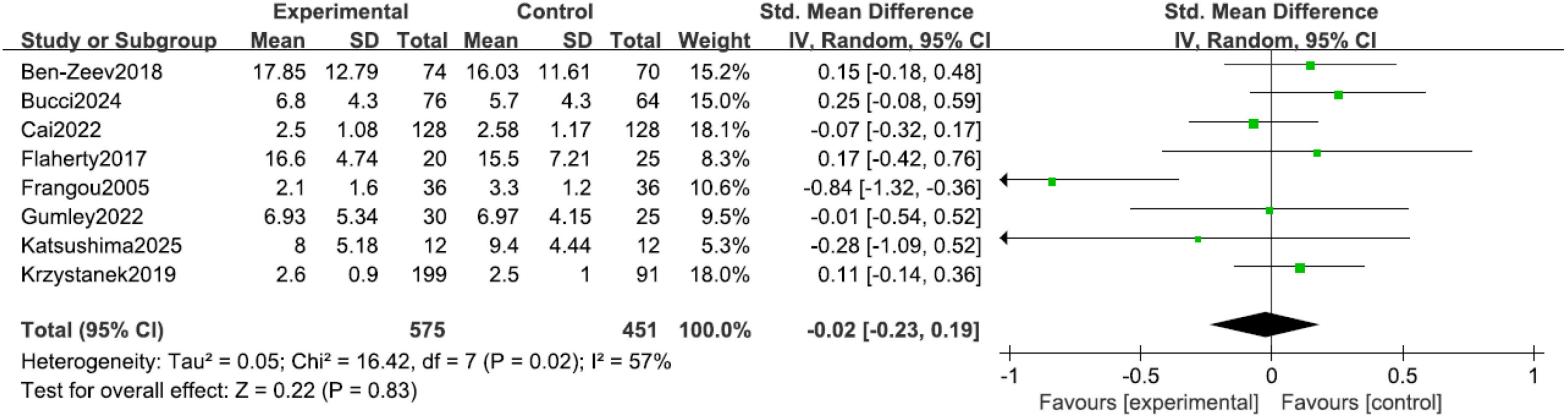
Forest plot of the meta-analysis for depressive symptoms.

Eight studies (9, 13, 14, 16–20) reported depressive symptoms in patients, with significant heterogeneity among the studies (I² = 57%, P = 0.02). Sensitivity analysis did not identify a clear source for this heterogeneity; consequently, an inverse-variance random-effects (IV, Random) model was adopted to provide a more conservative estimate of the effect. The results indicated no statistically significant difference in depressive symptoms between the two groups (SMD = -0.02, 95% CI = -0.23 to 0.19, P = 0.83), noting that a negative SMD would indicate improvement, as shown in **Figure 6**.

## Discussion

The results of this meta-analysis indicate that mHealth interventions can lead to a small but statistically significant reduction in positive symptom scores among patients with schizophrenia. Although the effect size is modest, its statistical significance should not be overlooked. These findings suggest the potential efficacy of mHealth in managing psychotic symptoms, which may operate through several synergistic mechanisms: first, continuous medication reminders and adherence feedback help stabilize drug plasma concentrations, thereby preventing symptom relapse at the biological level; second, digital courses based on CBT principles assist patients in acquiring coping skills, such as recognizing and challenging delusional thoughts and reducing reactivity to auditory hallucinations, thereby alleviating symptom-related distress and interference (20); finally, the ecological momentary assessment and intervention (EMAI) feature enables patients to receive real-time support at the onset of symptoms, overcoming the temporal and spatial limitations of conventional treatments and enabling more timely management (21). However, the GRADE assessment indicates that the certainty of this evidence is low, primarily due to risk of bias and imprecision. This suggests that the true effect might differ from this estimate. These findings provide preliminary evidence for the effectiveness of mHealth as an adjunct to conventional treatment in managing core psychotic symptoms in schizophrenia.

The results of this meta-analysis indicate that mHealth interventions did not significantly alleviate depressive symptoms in patients with schizophrenia on average (SMD = -0.02, P = 0.83). However, this null overall effect was observed alongside substantial heterogeneity (I² = 57%), suggesting that the impact of mHealth is not uniform and may be moderated by important clinical and methodological factors. The neurobiological complexity of depression in schizophrenia, which may involve distinct pathways such as cingulate cortex dysfunction as identified in neuroimaging studies (22), likely requires more targeted interventions than those offered by generic mHealth tools. In line with this, a recent feasibility trial of a comprehensive digital health tool found no significant effect on depressive symptoms, underscoring the challenge of effectively addressing this complex comorbidity within current digital frameworks (23). Furthermore, the considerable variability in intervention modalities is a likely key contributor to the heterogeneous findings. For instance, simplistic SMS reminders for medication adherence would not be expected to directly ameliorate depressive cognitions, whereas a targeted, interactive CBT program delivered via videoconference might. This spectrum of intervention complexity and therapeutic target, all categorized under ‘mHealth’, inherently generates diverse outcomes. The failure to account for these effect modifiers, combined with variations in follow-up duration, plausibly explains the statistical heterogeneity. Therefore, the null average effect should not be interpreted as a definitive lack of efficacy, but rather as an average of potentially positive, null, and even negative effects across different contexts and intervention types. This lack of average efficacy may be further understood through specific psychological mechanisms. Depression in schizophrenia often involves transdiagnostic processes such as rumination and defeatist beliefs, which generic mHealth CBT modules may not sufficiently target (24). Moreover, the therapeutic alliance—a key predictor of outcomes in depression treatment—is challenging to establish and maintain in fully digital interventions, potentially limiting their potency in addressing core depressive features like hopelessness and anhedonia (25). This underscores the need for future trials to pre-specify and target patient populations based on depressive symptom profiles and to employ more homogeneous, theory-driven intervention approaches. Future research should focus on developing more targeted modules specifically designed to address the psychosocial mechanisms of depression and consider adaptive intervention designs that provide personalized support based on patients’ real-time emotional states, thereby more effectively managing this complex and distressing comorbidity.

Beyond the uncertain effects on depression, this meta-analysis reveals a critical safety signal demanding heightened vigilance: compared to the routine care control group, schizophrenia patients receiving mHealth interventions exhibited a significantly higher incidence of adverse events. The absence of heterogeneity among studies indicates that this risk is consistent across different types of mHealth interventions, strengthening the reliability of the findings. Notably, the GRADE evaluation rated the certainty of this evidence as low, partly due to potential detection bias—a point that aligns with the following discussion. This discovery contradicts the common assumption that digital interventions are “harmless” or “low-risk,” necessitating a serious re-evaluation of the potential risks associated with mHealth applications in this vulnerable schizophrenia population.

The reasons for the increased risk of adverse events are likely multifaceted. The most immediate explanation is differential monitoring intensity. A core feature of mHealth interventions is continuous or high-frequency remote monitoring. For instance, app-based systems repeatedly prompt patients to report symptoms, mood, and medication adherence. This intensive self-monitoring may lead to the systematic recording and reporting of minor adverse events—such as transient anxiety exacerbation, sleep disturbances, or mild somatic discomfort—that might be overlooked in routine care (9). In contrast, the control group receives only standard outpatient follow-ups, where adverse event reporting relies more on patients’ spontaneous complaints or clinicians’ incidental findings, potentially leading to under-reporting. Therefore, the elevated odds ratio (OR) might partially reflect the additional information captured by the mHealth intervention as a more sensitive “monitoring system,” rather than a true increase in absolute harm. We had planned to conduct a sensitivity analysis restricted to studies with comparable active monitoring measures in both arms to help distinguish true harm from surveillance bias. However, due to the limited number of included studies and significant heterogeneity in how adverse events were monitored and reported across these studies, it was not feasible to identify a homogeneous subset of ‘actively monitored’ studies. Consequently, this analysis could not be performed. This underscores the necessity for standardized adverse event reporting in future trials. It is noteworthy that the GRADE evaluation also rated the certainty of evidence for the increased incidence of adverse events as low, partly due to detection bias from the lack of blinding, which aligns with our discussion on differential monitoring intensity. However, surveillance bias cannot fully account for the increased risk. The potential for mHealth interventions themselves to lead to iatrogenic effects must be seriously considered. For some patients, constant symptom monitoring and self-focused attention could translate into a state of “hyper-vigilance,” potentially exacerbating illness-related anxiety and stigma. When applications prompt users to focus on their mental health status, it may unintentionally reinforce their “patient” role, triggering or intensifying distress (26). Furthermore, technology-related issues, such as application malfunctions, data transmission problems, or frustration with device operation, can introduce new stressors. Particularly for patients with paranoia, concerns about data privacy or fear of “being monitored” may induce anxiety and mistrust, constituting unique adverse events (27). The increased risk of adverse events likely results from the combined effect of surveillance bias and potential iatrogenic risks. Future mHealth research and clinical applications must prioritize safety, establishing robust real-time risk monitoring and early warning systems. It is essential to ensure that while providing convenient interventions, necessary human supervision and immediate support protocols are in place to maximize the benefit-risk ratio.

This study has several limitations. First, the small number of included studies and their limited sample sizes reduce statistical power. Second, the restriction of our search to English and Chinese languages and the exclusion of unpublished literature may have introduced language and publication bias, limiting evidence comprehensiveness. Third, the scope of outcomes was narrow, omitting critical domains like negative symptoms, cognition, and psychosocial functioning, which are vital for recovery. Fourth, the generally short follow-up periods preclude conclusions on long-term efficacy and safety. Fifth, the potential for performance bias is high, as participants in digital trials are likely more motivated and digitally literate than the general schizophrenia population, affecting generalizability. Sixth, a key limitation of the adverse event analysis lies in the inconsistent reporting across original studies regarding the definitions of adverse events, monitoring intensity, and classification (e.g., by severity or type). This inconsistency precluded more in-depth subgroup analyses. Furthermore, as discussed, we were unable to perform sensitivity analyses to fully rule out the potential impact of surveillance bias on the results. Furthermore, this systematic review was not preregistered in a publicly accessible registry (e.g., PROSPERO). Although we adhered to a predefined internal protocol, the absence of preregistration introduces a potential for reporting bias, as the analysis plan and outcome selection were not fixed in advance. Finally, for the cluster-randomized EMPOWER trial (9), we could not adjust for the design effect due to missing intra-cluster correlation coefficients, which may have slightly overestimated the precision of its contribution; however, a sensitivity analysis suggested this did not alter the primary conclusions.

In summary, based on current evidence of low to moderate certainty, mHealth interventions for schizophrenia present a complex benefit-risk profile: they may offer a slight benefit for positive symptoms, show no significant effect on depressive symptoms, and may be associated with an increased detection or reporting of adverse events. This underscores the necessity for a cautious and individualized approach in their clinical application. Future research should prioritize: (1) identifying active intervention components via dismantling studies and developing targeted modules for depression and negative symptoms; (2) establishing robust risk-monitoring and management systems to optimize the benefit-risk ratio; and (3) conducting long-term effectiveness trials in real-world settings to identify patient subgroups most likely to benefit. Ultimately, through precise application that carefully considers individual differences and safety, mHealth has the potential to evolve into a safe and effective adjunct tool within the comprehensive management of schizophrenia.

## Data Availability

The original contributions presented in the study are included in the article/**Supplementary Material**. Further inquiries can be directed to the corresponding author.
